# Complete mitochondrial genome of *Oregonia gracilis* Dana, 1851 (Crustacea: Decapoda: Majoidea)

**DOI:** 10.1080/23802359.2021.1903362

**Published:** 2021-03-26

**Authors:** Sang-Hui Lee, Sang-Hwa Lee

**Affiliations:** National Marine Biodiversity Institute of Korea, Seocheon, Republic of Korea

**Keywords:** Complete mitochondrial genome, Decapoda, Brachyura, Majoidea, *Oregonia*

## Abstract

The complete mitochondrial genome of the majoid crab, *Oregonia gracilis*, was determined from a specimen collected in Korea. The mitochondrial genome is 15,737 bp long and contains 13 protein-coding genes (PCGs), 22 transfer RNA (*tRNA*) genes, and two ribosomal RNA (*rRNA*) genes. A maximum-likelihood phylogenetic tree based on the 13 PCGs of the mitochondria showed that *O. gracilis* is closely related to the genus *Chinoecetes*. The complete mitochondrial genome of *O. gracilis* provides valuable information on the mitochondrial evolution of majoid crabs.

Majoid crabs are one of the most diverse superfamilies of crabs, and 907 species from 189 genera belonging to six families have been reported worldwide (Davie et al. 2015). Because the majoid crabs include various species, their systematics and phylogeny are complicated (Griffin and Tranter 1986; Ng et al. 2008). Among these crabs, the genus *Oregonia* contains two species, *O. bifurca* and *O. gracilis*, of which only *O. gracilis* has been reported in Korean waters (Kim 1973; Ko and Lee 2015). No study has yet described the complete mitochondrial genome of either species of the genus *Oregonia*. Therefore, in this study, we determined the complete mitochondrial genome of *O. gracilis* for the first time.

An *O*. *gracilis* specimen was collected with a local fisherman’s fish trap in the coastal waters around Busan, Republic of Korea (geographic location: 35°9′13.29′′ N 129°7′40.45′′ E) on 11 April 2019. The voucher specimen (MABIK CR00247474) was deposited at the National Marine Biodiversity Institute of Korea. The genomic DNA was extracted from the pereiopod of the specimen with the DNeasy Blood & Tissue kit (Qiagen, Hilden, Germany). The complete mitochondrial genome was sequenced with long-range PCR. First, the *COXI* and large rRNA (*lrRNA*) – small *rRNA* (*srRNA*) genes were amplified, and then the gaps between partial genes and long fragments were amplified with the long-range PCR technique, using the newly designed primer sets shown in Supplementary Table 1. The PCRs were performed in a 20 μL volume containing 17 μL of distilled water, 1 μL of each primer (5 pM), and 1 μL of *O. gracilis* genomic DNA and AccuPower ProFi Taq PCR PreMix (Bioneer, Daejeon, Korea). The PCR thermal cycling conditions were: 95 °C for 5 min; 35 cycles of 95 °C for 20 s, 55 °C for 30 s, and 68 °C for 10 min; with a final extension at 68 °C for 10 min. To determine the complete circular mitochondrial genome, the two PCR amplicons were directly sequenced with the primer walking method using the BigDye Terminator version 3.1 Cycle Sequencing Kit (Applied Biosystems, Foster City, CA) and DNA Analyzer 3730xl (Applied Biosystems, Foster City, CA) at AquaGenTech (Busan, Republic of Korea). The genes were annotated according to their locations in the complete mitochondrial sequence with MITOS (Bernt et al. 2013).

The complete mitochondrial genome of *O. gracilis* is 15,737 bp long (GenBank accession no. MW367446). It encodes 37 genes: 13 protein-coding genes (PCGs), two ribosomal *RNA* genes (rRNAs), and 22 transfer *RNA* genes (tRNAs), with a noncoding region of 716 bp. The mitochondrial genome’s base composition is 37.2% A, 16.1% C, 8.5% G, and 38.1% T, with a G + C content of 28.1%. The lengths of the *lrRNA* and *srRNA* genes in this species are 1,311 and 990 bp, respectively, whereas the length of the transfer RNAs identified range from 61 to 77 nucleotides. There is a putative control region (716 bp) between lrRNA and srRNA, as in other majoid mitochondrial genomes. There are three types of PCG start codons, ATA (*NAD1*), ATG (*ATP8*, *CYTB*, *COX1*–*3*, *NAD2*, *NAD4*, *NAD4L*, and *NAD5*), and ATT (*ATP6*, *NAD3*, and *NAD6*). TAA (*ATP6*, *NAD1*, *NAD3*, *NAD4*–*6*, and *NAD4L*), and TAG (*ATP8*, *CYTB*, and *NAD2*) are used as the stop codon, whereas three PCGs (*COX1*–*3*) have incomplete stop codons (T).

The complete mitochondrial genomes of 13 species of crab were used to establish the phylogenetic relationships of *O. gracilis*. The complete mitochondrial genomes of 11 section Eubrachyura species (five majoids, two portunoids, two xanthoids, and two potamoids) were retrieved from GenBank, together with that of a homoloid species, *Homologenus malayensis*, from the section Podotremata, which was used as the outgroup. A phylogenetic tree was reconstructed based on the 13 concatenated PCG sequences of the species using the maximum likelihood (ML) method with RAxMLGUI version 2.0 (Edler et al. 2020). Bootstrap values were calculated from 10,000 replicates. The results showed that *O. gracilis* clustered with the species of the superfamily Majoidea, and that it was most closely related to the clade containing *Chinoecetes japonicus* and *C. opilio*, with high bootstrap values (Figure 1).

**Figure 1.**
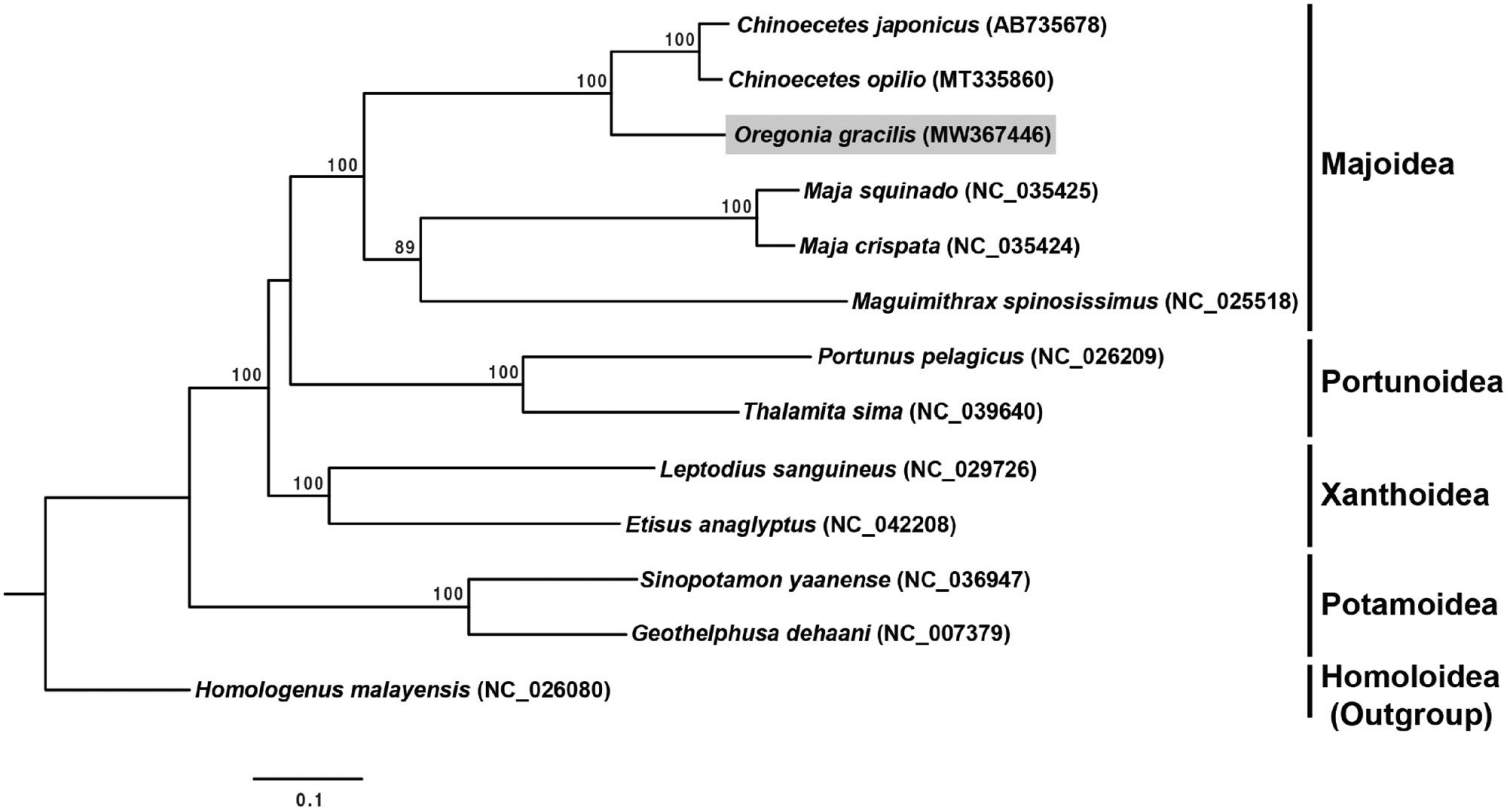
A phylogenetic tree constructed from 13 concatenated mitochondrial protein-coding genes of each species using the maximum likelihood (ML) method, with the GTR + G + I model. The position of *Oregonia gracilis* was examined in this study. Bootstrap values were calculated from 10,000 replicates, and those >70% are indicated at the base of each node.

## Data Availability

Mitochondrial genome sequence can be accessed *via* accession number MW367446 in GenBank of NCBI at https://www.ncbi.nlm.nih.gov. The associated BioProject, SRA, and Bio-Sample numbers are PRJNA687105, SRR13285880, and SAMN17132501, respectively.
